# “We are mothers, sisters, and lovers too”: Examining young Black women's experiences navigating sex and sexual health

**DOI:** 10.1002/ajcp.12753

**Published:** 2024-05-12

**Authors:** Natasha A. Darko, Ciann L. Wilson, Vanessa Oliver

**Affiliations:** ^1^ Department of Psychology, Faculty of Science Wilfrid Laurier University Waterloo Canada; ^2^ Faculty of Liberal Arts, Child and Youth Studies Wilfrid Laurier University Brantford Canada

**Keywords:** African, Black women, Caribbean and Black, community‐based participatory research, sexual health

## Abstract

In Canada, there is a lack of research that addresses the sexual health and well‐being of African, Caribbean, and Black young women. This paper aims to gather perspectives of young Black women to address the social contexts of how young Black women navigate issues related to sex and sexual health. Young Black women experience unique dynamics in navigating their sexualities and sexual healthcare. The nuanced experiences stem from social contexts with historical underpinnings, such as the perception of Black women's bodies, Black identity, gender roles, and sexual double standards. This Community‐Based Participatory Research study (*N* = 24) utilized focus groups to examine young Black women's experiences navigating sexual health. Employing a thematic analysis, participants identified four themes representing their narratives of navigating sexual health. The themes included the perceptions and hypersexuality of Black women's bodies, navigating sexual double standards and gender roles as Black women, diverse Blackness, and migration experiences concerning sexual health and surveillance of Black women's bodies. This paper is intended to add to scholarly discourse and will include practical strategies for use by researchers and community practitioners in sexual health within the Black community.

## Keypoints

- Provides a narrative to the social contexts in which young Black women navigate sexual health.
- Fills a gap in the literature by addressing the social factors that affect young Black women.
- The narratives of Black women are rarely included when talking about sexual health.
- Young Black women often navigate between embracing and rejecting perceptions of.
- Black womanhood.

## INTRODUCTION

Edwards and Coleman (2004) define sexual health as “physical, mental, and social well‐being.” Sexual health goes beyond the absence of disease or health outcomes and encompasses the possibility of pleasurable, comfortable, and safe sexual experiences. This study embraces the Edwards and Coleman (2004) definition of sexual health to explore the social contexts that impact young African, Caribbean, and Black (ACB) women.^1^ Women's^2^ ability to navigate sexual health. This definition provides a more holistic definition of sexual health, including sexually transmitted diseases (STIs), HIV prevention, sex positivity, and bodily autonomy.

Despite being a small proportion of the Canadian population, Black communities experience disenfranchisement in many aspects. This stems from Canada's history of systemic anti‐Black racism. Anti‐Black racism is perpetuated through the legacy of the current social, economic, and political marginalization of African Canadians in society, such as the lack of opportunities, lower socioeconomic status, higher unemployment, significant poverty rates, and overrepresentation in the criminal justice system (Mullings et al., 2016). The trend of economic, political, and social marginalization continues within the healthcare system, where Black communities report experiencing a lower quality of care (Fante‐Coleman et al., 2022) and are overrepresented in poorer health outcomes, such as diabetes, heart disease, stroke, and HIV/AIDS (Kaul et al., 2011; Veenstra & Patterson, 2015).

This trend continues for Black women and HIV, who accounted for 42% of newly reported HIV cases in Canada in 2019 and, in 2020, accounted for 19.8% of newly reported HIV cases (Owino et al., 2022). Research also shows that Black youth are diagnosed with HIV and AIDS at increasingly younger ages than other racial groups (Public Health Agency of Canada, 2012). Existing research focuses on individual risks for Black women, often overlooking the nuanced social contexts that Black women face when accessing sexual health programming and services. The gap in literature impedes the provision of relevant sexual health programming and interventions for young Black women. The imperative nature of this exploration is highlighted by the disparities above in HIV statistics for young Black women. This article aims to include Black women's narratives on the larger social contexts they navigate in sexual health. This addresses the gap in the literature by providing perspective on the social factors that affect young Black women's experiences navigating sex and sexual health.

The question that guided this research process is: What insight can be gained from centering young Black women's (ages 16−29) voices in research about their sexual health? Through engagement with experienced researchers, background research, and personal reflection, a Community‐Based Participatory Research (CBPR) methodology was utilized to guide this study. CBPR prioritizes reciprocal equitable partnership between scholars and community partners involved in a research process. Toward this end, community members, organizational representatives, and researchers are involved in informing decisions in all aspects of the research (Israel et al., 2005). Researchers have used CBPR in marginalized communities to successfully address a variety of health‐related issues, including tobacco use, cancer prevention and control, elder abuse, youth wellness, genetic problems, environmental exposures, and HIV/AIDS (Baffour & Chonody, 2009; Daley Maksosky et al., 2010). Due to the historical and contemporary unethical treatment of Black people in research, a CBPR approach was deemed most appropriate by the research team and various stakeholders.

## LITERATURE REVIEW

Contemporary readings in Western society of Black women's bodies and their sexualities pre‐date the Trans‐Atlantic slave trade. It is crucial to examine the historical context that influences and continues to inform the gendered and sexual scripts that confine Black women's sexualities and sexual health today. From the onset of colonialism, when European colonizers feverently exploited the peoples, land, and animals of the African continent, they perceived Black people as evolutionarily degenerate compared to the European man, with sub‐human capacities (Story, 2010). This opinion that Black people were evolutionarily closer to animals than humans justified the mistreatment of Black people during the era of the Trans‐Atlantic slave trade. Over 20 million Black African people were estimated to be taken from their homelands and sold into slavery throughout the Americas (Noonan et al., 2016).

A common thread linking all stereotypes about Black women is how they portray them as anything but human. For instance, the “Strong Black Woman” stereotype—as most recently articulated through the #Blackgirlmagic term, has inherently labeled Black women as ever‐resilient, inhuman beings that can withstand insurmountable forms of suffering and pain, despite the multiple layers of discrimination, violence, and oppression directed at them within a society that often devalues and fails to protect Black women (Chavers, 2016). We see remnants of this thinking among healthcare providers, who have been reported to retain historically rooted racist stereotypes that Black people have higher pain tolerance than other racial or ethnic groups (Hamid, 2023). This often manifests in young Black women being raised to disregard their own needs and perspectives, instead focusing on the needs of those around them (Aniwayo et al., 2022). These stereotypes continue to affect perceptions of Black women, and in turn, Black women internalize these stereotypes, which can have deleterious impacts on their mental health and sense of self.

Another relevant stereotype about Black women that is pervasive and also stems from colonialism is that of the hypersexualized Black woman or the “Jezebel.” Often hypersexualization is misinterpreted to imply an “overactive” sex life; however, it is defined as the over‐sexualization of expressions, mannerisms, and dress codes (Cottais & Louvert, 2021). During the enslavement era, the simultaneous animalization and over‐sexualization of Black women made them targets for sexual violence from their captors. They painted them as “dangerous,” “dirty,” and thus a risk for promiscuous animal‐like sexual proclivities that could transmit venereal diseases amongst White settler communities (Feder, 2007). Positioning Black women in this way effectively demarcated them as different from the standards of womanhood, purity, innocence, and femininity afforded to White women. These scripts can be seen in the contemporary examples of how the English Rose, Kate Middleton, was juxtaposed with the bi‐racial divorce, Meghan Markle.

A study on Black women's representation in the media conducted by Johnson (2015) highlighted that 90% of magazines with Black women on the cover showed images of Black women being hypersexualized concerning the clothing and make‐up they wore, in addition to the positions and poses they were asked to strike for the people behind the camera—who are often White men. Additionally, Turner (2011) found that in music videos, Black women were likelier to appear in provocative clothing than women of any other racial background. These contemporary images perpetuated by the media build upon historically rooted anti‐Black sexism and showcase Black women as hypersexual, or sexually available for men (be that consensual or not), and simple‐minded (or unintelligent) (Collins, 1990). An example is the Wet Ass Pussy (WAP) video by Cardi B and Megan Thee Stallion. The video is filled with overtly sexual images of both rap stars and sexually charged lyrics that focus on the valuation placed by both women on their sexual skills in the bedroom and their genitalia. The video was acclaimed by many and shrouded in controversy that surrounded it because of the sexual content. The video brings the viewer to a hidden Hollywood mansion. It is evident that although set in a fancy mansion associated with wealth and affluence, these women are, as the lyrics say, the mansion is to be understood to be more of a brothel. Cardi B, Megan the Stallion, and their proponents have highlighted that the song is about Black women reclaiming and owning their sexuality through women's sexual liberation (Turner, 2011; Weekes, 2020). It is essential to highlight that in the early days of hip hop in the 80's and 90's, Black female rappers did not have stylists and an industry team behind them. MC Lyte, Queen Latifah, Sista Souljah, Lisa Left Eye Lopez, Missy Elliot, and others relied more heavily on their lyricism and esthetic as the stereotypical “tomboy” to sell records and carve out unique and indelible spots in the culture for themselves.

### Concepts that inform this work

Black feminists have been exploring the meaning of being a Black woman for decades (Hill Collins & Bilge, 2016). During the 1960s and 1970s, Black women developed an *intersectional* analysis within social movements. An example was Frances Beal's essay, “Double Jeopardy: To be Black and Female,” published in 1969. The piece laid out the patriarchy within the Black Power Movement and the racism within the White Feminist Liberation Movement (Beal, 2008), ultimately leaving Black women out. Scholars often use intersectionality to question society's ambiguous relationships with Black women, who were not perceived as feminine as White women but were simultaneously expected to be subordinate and subservient to Black men. Black women's work and voices laid the groundwork for what became known as intersectionality (Brah & Phoenix, 2004; Crenshaw, 1989).

Intersectionality is crucial to health research because it embraces the complexities of understanding health inequities (Hankivsky & Christoffersen, 2008). The theoretical framework of intersectionality explores the unique experiences of Black women's multidimensional identity. According to Bowleg (2012), the central tenets of intersectionality are that social identities cannot be reduced to one entity, and they are multiple and intersecting; people from marginalized and oppressed groups are the center of intersectionality; multiple social identities at the micro‐level intersect with community, society, and structural factors to produce health inequities. Using an intersectional lens is essential to address the various forms of oppression that Black women face to gain a clearer perspective of the broader social contexts that impact sexual health and also to provide relevant solutions in helping young Black women achieve positive outcomes when it comes to sexual health and sexual well‐being. Utilizing an intersectional approach in sexual health programming and interventions acknowledges that the experiences of Black women cannot be reduced to a single entity.

### A section on Black women's sexual health^3^

Black women are at notably higher risk for contracting an STI in their lifetime despite engaging in less risky sex than their counterparts of other racial groups (Oser et al., 2017). In 2020, Black women accounted for 42% of all women newly diagnosed with HIV in Canada. Gonorrhea rates in young women in England are highest among Black women (Solomon et al., 2022). Black girls aged 15−19 are 4.5 times more likely to contract chlamydia in the United States (Centers for Disease Control and Prevention, 2019). In 2019, Black teens were twice as likely to get pregnant and give birth than their White counterparts (Martin et al., 2021). Compared to other racial groups, ACB young women are more likely to report poor healthcare experiences and unfair treatment and, in turn, were the least likely to access information and resources from any healthcare source, including sexual health services (Flicker et al., 2009). Despite the research highlighting the sexual health consequences (HIV, STIs, and pregnancy) for Black women, sexual health programs focus on risk factors, not the perspectives of Black women and their sexual health.

## RESEARCH OBJECTIVES

The larger *Sista2Sista* project is a research project focused on adapting the Health Improvement for Teens (HIP Teens) Intervention from the American to the Canadian context. HIP Teens is an evidence‐based sexual health risk reduction intervention for adolescent girls that enhances knowledge, increases motivation, and teaches the behavior and skills needed to reduce pregnancy, HIV, and STI risk (Morrison‐Beedy et al., 2013) for application in the Canadian context. Through conversations with young ACB women, the authors explored their journey of navigating sex and sexual health instead of focusing on HIV statistics and risk factors. This paper's primary aim is to gather the perspectives of young Black women to shed light on the social context within which they navigate matters related to sex and sexual health. The secondary research objectives of this paper include: (1) address a gap in the literature to provide a deeper understanding of the primary social factors that influence young Black women's decision‐making and behaviors related to sex and sexual health, (2) include practical strategies for use by researchers and community practitioners who work in sexual health, namely Black sexual health.

## METHODS

### Advisory committee

Given that the *Sista2Sista* project was a CBPR project, we engaged an advisory committee in informing the work. The Youth Advisory Committee—YAC comprised seven youth Black people across the Greater Toronto Area (GTA). The YAC was open to Black people regardless of gender because the perspectives of all Black youth are essential to informing an adequate sexual intervention that focuses on the sexual health of Black youth. The YAC was recommended by research team members and identified through the vast networks of community partners, such as the AIDS Committee of the York Region, the AIDS Committee of the Durham Region, and 360 Kids. Each organization was involved in the larger *Sista2Sista* project. The YAC assisted with the recruitment of participants and extensive promotion, provided their insights on the design of the posters and recruitment materials, and provided insight into the state of sexual and reproductive health in Ontario. The YAC was strategically included in the research approach to extend this research study beyond the bounds of academia, it was important that the research team specfically engaged with communities members in this research.

### Participants

The requirements to participate in the focus groups were that the participants were young Black women—identifying individuals indicating an interest in trans women participants as well. Participants had to be between the ages of 15−29 and reside in the GTA. Recruitment occurred in many forms, including posters in Black‐focused community organizations (i.e., Black Coalition for AIDS Prevention), community events (i.e., Afrofest), and in‐person at local health centers, community centers, high schools, and shopping malls. The collaborating community organizations were also approached to access young Black women at their sites and centers to participate in the research project. Online platforms, such as social media and email promotion, were also employed for recruitment. Online flyers were distributed to Black community organizations through various email listservs throughout the (GTA). In total, 40 young women were interested in joining the focus groups and participating in the study. However, six people were excluded because they needed to meet the age requirements. Another three people were excluded from participation because they did not reside in the GTA. One participant was excluded because they did not identify as a woman or a trans woman. The remaining six participants had a variety of scheduling conflicts and could not join the focus groups.

A final sample was obtained of 24 young Black women participants (*N* = 24), with a mean age of roughly 24. There were two focus groups held in the summer of 2019. The first focus group had 13 participants (*n* = 13), and the second had 11 (*n* = 11). The sample consisted of young Black women between 16 and 29. All participants identified as ACB. Twenty‐three participants identified as heterosexual, and one participant identified as bisexual. All participants identified as cis‐gendered women, reflecting a possible limitation in our study design that is unpacked in our limitations section.

### Procedure

#### Data collection methods

##### Demographic survey

Participants were provided brief, anonymous demographic surveys at the beginning of each focus group. Participants were asked about their age, where they lived, their sexual orientation, gender identity, and ethnicity. The surveys were accessible and jargon‐free.

##### Focus groups

In garnering the perspectives and lived experiences of ACB youth in the GTA, we aimed to recruit 25 young Black women for the focus groups. Ultimately, only two focus groups were necessary to meet our participant quota. A focus group guide was used to initiate a discussion about the experiences of Black young women accessing sexual health services and information in the GTA. Questions were co‐created by the first two authors with the goals of the broader *Sista2Sista* project in mind. Participants were asked questions regarding (a) sexual health, (b) access to sexual health services, (c) sexual health education, and (d) preferred sexual health resources. The research questions were tailored to align with the objectives of the larger intervention project.

To facilitate a fulsome discussion about sexual health for Black women, participants were asked questions such as: (a) When I say the word sexual health, what comes to mind? (b) What are the most important sexual health issues facing young, Black women in the GTA? (c) What sexual health programming is available to you to discuss these issues of sex, sexual health, STIs, and so forth? (d) How would you like to receive information on sexual health? (e) What is the best way to engage Black youth and get them involved in sexual health issues?

Employing a qualitative research method was necessary because it allowed the participants to engage in meaningful discussions about their views and experiences. Focus groups encouraged discussions about sexual health services and programming for young Black women. Focus group methodology has explored various issues surrounding adolescent sexual health (DiCenso et al., 2001). According to Krueger and Casey (2014), open‐ended interview questions allow participants to talk freely and form discussions about topics such as sexual health education and services.

Two 90 min focus groups were conducted and facilitated by two of the authors, both Black and cis women‐identifying. The first and second authors facilitated the first focus group. The first author facilitated the second focus group alone. Ground rules were discussed at the beginning of the focus group sessions. All participants were given an informed consent form at the beginning of the focus groups. This form was explained and signed before the audio device was turned on. Due to the sensitive nature of sexual health and HIV, participants were encouraged NOT to share any personal information they did not feel comfortable disclosing publicly (i.e., HIV status) with others in these group sessions. Facilitators ensured that no participant spoke more than the other or spoke over others. This was done through the facilitators intentionally moderating the discussion and probing questions of participants who spoke less.

### Data analysis

#### Qualitative analysis

Data analysis took place after all focus groups had been completed. Focus groups were audio‐recorded and transcribed verbatim. The transcripts from the focus groups were analyzed using a thematic analysis framework for common themes using NVivo 11 qualitative data analysis software. This process was completed using Braun and Clark's (2006) six‐step process. A codebook was co‐created by the first two authors. Potential codes and themes were selected after each focus group and were narrowed down until they were clearly defined. Due to the time constraints surrounding the project, when the codebook was created, it was presented to the members of the YAC during a quarterly meeting for their approval and edits. The members of the YAC provided insightful feedback, such as the inclusion of the theme of sexual double standards. To ensure that data from each focus group was represented, quotes taken from each focus group were clearly labeled. The first author analyzed the data. The first author analyzed the data along with another member of the research. Due to varying schedules and time constraints on the project, there was not a discussion of YAC members being involved in data analysis. It is important to acknowledge that CBPR is a spectrum and should be adjusted to fit the various research contexts (Spears Johnson et al., 2016). However a potential limitation is that certain insights and perspectives could have been missed because peers could provide depth to the existing findings.

## RESULTS

This study aimed to identify the social context, lived experience, and realities of the sex and sexual health of young Black women in the GTA. Another goal of this project was to provide a space for young Black women to discuss their experiences navigating sexual health and to allow participants of similar backgrounds who do not know each other to engage in discussions and enable new opinions to form. In each focus group, participants had candid conversations regarding their sexual health and lived experiences as Black young women living in the GTA.

Participant conversations fell into one of four significant themes representing their narratives of navigating sexual health. The themes were: perceptions and hypersexuality of Black women's bodies, navigating sexual double standards and gender roles as Black women, diverse Blackness, and migration experiences concerning sexual health and surveillance of Black women's bodies. The themes emerged from the codebook and subsequent analysis. Each theme and subtheme will be explored further below.

### Perception and hypersexuality of Black women's bodies

#### Perceptions of Black women's bodies

Participants discussed how Black women's bodies are perceived and imagined as unfeminine and worthy of protection in the way White women's bodies might be, and they spoke to the implications of this in healthcare settings.

One young woman said:

It almost feels like Black women's bodies are not seen as something to care for, nurture, and handle in a safe and caring way… You know it's not this promiscuous, tough, rough sexualized image of Black women. We are also mothers and sisters. Putting those images out, maybe when we do go to the doctors or healthcare providers, we will be given respect, we will be treated kindlier, and we will be listened to. And maybe putting those images out will help with that too. (Focus Group 1)

This quote speaks to the narratives surrounding Black women's bodies. The young woman also mentioned that Black women are more than just their bodies, that Black women have multiple roles, such as being mothers, sisters, and lovers, and are fully human. These sentiments were echoed throughout the focus groups. Participants also spoke about their bodies and decisions around their bodies not being their own. Young Black women also talked about the lack of autonomy that Black women often feel regarding their bodies. Another young woman said:

First off, I'd like White men to stop making decisions about my Black female body, but in addition to that, I'd like just once to walk into a conversation and have that start with, you know, whatever you tell me, I'm going to believe you. And I'm going to work with you to find out a solution. It's not on you to fight to advocate for yourself, to beg for a service, or to beg for support. This is us together, going on this journey. I think that as a starting point would change a lot of conversations. (Focus Group 1)

This quote echoes the participants' opinions on the notion that Black women do not get to make decisions about their bodies. It also speaks to the need to take Black women's needs seriously and to create solutions working with Black women for Black women. Throughout the focus groups, the young women often spoke of their feelings and experiences being invalidated by the larger society. This is echoed when Black women continually must advocate for their health and access to related services and supports. Participants also discussed the perceptions of the sexualities and associated sexual health of Black women.

#### The hypersexualization of Black women's bodies

Participants also spoke about the hypersexualization of Black bodies through various outlets, including the media. They spoke about how the stereotypes surrounding Black women and their bodies have led to stigma when accessing health services. One participant noted:

I feel like this stigma that Black women face that's mostly based on how Black women are seen in many ways, in the media, and in everyday lives. Black women are stigmatized to be viewed as women that are very sexually active, and I guess sexually expressive, and they're comfortable with pregnancy… they'll [doctors]automatically assume you are pregnant, you have an STD because of the things that they see outside of the world with regards to Black women. (Focus Group 2)

This quote outlines how the stigma about Black women and their sexuality has led to the assumption that Black women are more likely to have STIs and be pregnant. The discussions in the focus groups spoke to how Black women were often misdiagnosed due to assumptions about Black women's sexuality. The young women also spoke of people policing how they dress and their behaviors to prevent the perception that they are sexually promiscuous or hyperactive.

One participant said:

… the double standard, I find that too, comes from a place of fear; women, Black women especially, are sometimes hypersexualized. My mom specifically is very cautious about like what you are wearing, what you are saying, who you are talking to because people are not just going to paint you as a sexual person. They'll paint you as a Black sexual woman, and that is somehow worse. I find that sometimes, even if I wanted to express my sexuality, I couldn't because it would be perceived as being promiscuous or not that even being promiscuous is a bad thing. (Focus Group 1)

This quote outlines the hypersexualization of Black women's bodies from a place of fear. This participant spoke about how their mother wanted to protect them from being labeled as sexual beings at a young age and how the repercussions of this labeling are negative. This quote demonstrates that if a Black woman is perceived to be sexual, it is a negative thing. It further showcases that Black women's bodies are often policed by various sources, starting with other Black women closest to them. Thus, furthering the notion that Black women are judged more harshly than their White counterparts in expressing their sexuality.

### Navigating sexual double standards and gender roles as Black women

#### Sexual double standards

Participants discussed how men and women are held to different standards concerning sex and sexuality. They spoke about how men in their lives were educated about safe sex and where to access condoms. In contrast, they, as young women, were advised against having sex or participating in sexual activity. One young woman said:

…my parents are of an African background too. With my mom, it was always don't have sex. It was never, why, and this is how, it was always don't do it, don't get pregnant type of thing. I do remember one instance where she spoke to my brother, and she was asking him if he knew about condoms and stuff and where to get it. I think a lot of it, too, is the double standards, where it's okay for a boy child, it's okay as long as he is using condoms, but daughters cannot have sex. (Focus Group 1)

This quote showcases the stark differences in how young women and men are raised and socialized to think about sex. The men are permitted to have sex as long as they are using condoms and being safe, but the young women are shamed in discussions about sex. Participants also spoke about how it is not a generational issue and that their partners believe in upholding the same standards for their male child. Another young woman said:

Even now, even talking to my boyfriend, I do the same thing. He's like, no, we have a daughter, we can't have any boys come over, but if it's the son, yes, you know, make to give them some condoms. I'm just like, why the double standard? I think that's part of it too. (Focus Group 1)

The quotes above showcase the dichotomy between men and women regarding sexuality and sexual health. Participants noted that men were permitted to talk openly about sex and to have sex; however, with young women, sex was viewed as taboo and not a topic of discussion. These gendered double standards also relate to the gender roles that exist between men and women.

#### Gender roles

Along with double standards related to gender, young women also spoke about the pervasiveness of gender roles in their everyday lives. They spoke of the societal pressures of being a young Black woman and the expectation to seek marriage and to be the “ideal woman” for a partner. One young woman said:

I also feel it's the transition of life in university. I thought that was my time to have fun and be myself and do all these crazy things, but then, when I graduate, I'm adulting… This is what I shouldn't do. I should look for a husband…And, then, even ideals of relationships, he wants me to be this, or she wants me to be this, or I should please them this way by doing this. Yes, I think that's a huge issue too with health, sexual health if you want protection, but you don't want to make your partner feel uncomfortable, or if you're having a battle between that, you could be health‐conscious and your partner. (Focus Group 1)

This quote exemplifies the young women's feelings toward traditional gender roles, in which they felt that their time to have fun was during their postsecondary education. However, as soon as that ended, they were expected to become adults and to seek the socially expected ideals of relationships. This quote also speaks to the gender dynamics within sexual relationships, in which women feel they cannot negotiate condom use because it would appear that they do not trust their partners. Participants also noted that, in many relationships with male partners, a dynamic did not allow for negotiation, especially condom negotiation within heterosexual relationships. Due to this power imbalance between participants and their male partners, participants often faced sexual objectification and low self‐esteem. This dynamic was exacerbated when the male partner provided financial support.

One young woman noted:

Most of it, it's small things, and then, it works itself up to bigger things and situations, but still, at the end of the day, if you're forced to do something in exchange for something else, then that is still a level of prostitution, and I feel a lot of the youth don't understand that. You're so lucky; he does X, Y, Z, or, you know, right after, he's not giving me $300, but it's not a real, platonic relationship. This is happening; you're doing it in exchange, right? The different levels of it. (Focus Group 2)

Participants also noted that traditional Western gender roles were very much enforced in their daily lives. The quote above signifies that in a heterosexual relationship, when the male partner provides financial support, it is often seen as a romantic gesture. However, participants made mention of the lack of power that comes with such relationships, which, in turn, affects their ability to negotiate the timing and safety of sex and their sexual health.

### Surveillance of Black women's bodies

Participants also reported their Black bodies being watched continuously and surveilled. Participants spoke about their experiences of being over‐monitored, covertly or overtly, daily. This caused young women to feel like they were being judged more harshly than their non‐racialized counterparts. One young woman said:

I don't know. I feel when it's with people that are not necessarily my race, I feel it's a different kind of judgment versus if it's the ones that are kind of in the same culture. I don't know, that sounds kind of contradictory, but at the same time, it's you know what I mean. It sounds contradictory; I feel more comfortable around people that look like me versus being judged by people that have no idea of what's going on. (Focus Group 2)

This quote showcases the general observations made by the participants. They felt more comfortable with people who were of the same race. The young women felt the judgment from others outside of their race was harsher. The overall sentiment was that they would rather be judged by someone who looked like them than by someone who did not. However, some participants noted privacy concerns when meeting with someone of the same ethnic‐racial background because of the chance that their personal information would get back into the community. One participant noted:

I feel if it's people of the same culture, I'd be more hesitant. If it were a different culture, I'd probably be open a little bit more. I don't know. Some people give you that look or demeanor, and you look like every single auntie. (Focus Group 2)

Both quotes signify that no matter where Black women are, they are constantly surveilled or perceived that they are constantly being watched both by people who look like them and by people of other races. This makes it difficult for young Black women to access services and information regarding sexual health.

### Diverse Blackness and experiences of migration concerning sexual health

Participants also discussed how the Black community is often viewed as a monolith. However, the Black community has many different social locations and contexts. The common thread connecting participants was their Black identity. However, there is diversity in Blackness. One young woman said:

Although we're all Black folks in this room, there are different experiences of Blackness based on country of origin, culture, where you're situated, how long you've been situated in Canada, all of those things I think to matter a lot. (Focus Group 2)

This quote explains Blackness in Canada. Black people in Canada come from different cultural experiences, immigration experiences, and ethnic backgrounds. Often this is left out of the conversation about Blackness and who is considered Black. The experiences of every Black person differ based on their lived experience, and it is essential to acknowledge this. This also highlights Black women's full diversity, as well as being diverse in our humanity. Black women cannot be compartmentalized and lumped into categories.

## DISCUSSION

The results of this study demonstrate that there are diverse experiences of Blackness in the GTA, and these experiences often impact young Black women's experiences of socially expected gender roles, perceptions and stereotypes of Black bodies, and surveillance of Black women's bodies. These findings highlight that young Black women need sexual health programming and services to go beyond the “risky sexual behavior” narrative and to encapsulate the sociocultural and structural factors that contribute to Black women's experiences around sex and sexual health. Beyond the themes mentioned above, the overall purpose of this study was to contextualize the *Sista2Sista* study in the Canadian context. In the results section of this paper, participants candidly discussed several topics that highlighted their collective experience of being Black women and navigating sex and sexual health.

Participants expressed that, often, Blackness is seen as a monolith and that all Black people are assumed to have similar life experiences regardless of migration stories and positionality. Existing sociological research on Black identity does not discuss the nuances of being Black in North America (Dei, 2018), much less the experiences of living in Canada versus the USA. Place of birth, social class, migration history, or multiracial origins all impact experiences of Blackness. Research on Black identity often fails to focus on sociocultural identities (Charles et al., 2015). Additionally, a study conducted on first and second‐generation African and Afro‐Caribbean immigrants suggests that they do not consider themselves culturally homogeneous with Black people born in North America or multigenerational Black people in North America. Hence, they have different notions of the Black identity (Charles et al., 2015; Johnson, 2016; Warner, 2012; Waters et al., 2014). This study contributes to the literature by explicitly addressing the complexity of being Black within the context of sexual health. Based on the diverse experiences of Black young women, their ways of navigating sexual health can be very different. This study highlights that though Black women are diverse in many ways beyond ethnicity, socioeconomic status, and geographic location, there is a common thread that connects all the Black women in this study, the experience of racial discrimination. The findings of this study showcase the importance of considering intersectionality when designing sexual health programs and services targeting Black women (Flowers, 2018; Prather et al., 2018). Due to the intersecting identities of Black women, which include not only gender but also race and sexual orientation. Sexual health programming and services cannot be a “one size fits all” solution for Black women and their social locations.

Participants discussed how gender roles impact how young Black women are expected to uphold Euro‐western society's monogamous and heteronormative standard of seeking marriage and reproducing. Attitudes and behaviors toward sex are often based on culturally and societally determined gender roles (Nguyen et al., 2010). Gender norms influence what is deemed appropriate for men's and women's social and sexual interactions. Feminine gender role expectations may lead to a lack of communication about sex and sexuality (Cislaghi & Heise, 2019; Kachel et al., 2016). This is also rooted in patriarchy, which may lead to young women compromising their needs and being passive. The assertive masculine role and the passive feminine role, which are present in gendered sexual dynamics, are exacerbated by race. Young Black women often navigate between embracing and rejecting perceptions of Black womanhood (Collins, 2000). This means that Black women often work hard and do the extra work to liberate themselves from negative stereotypes imposed on them in a society that usually does not value Black womanhood. Though this study mainly focused on young Black women, it is also essential to include young Black men in these conversations. Due to the gender inequalities in the experiences of sex and sexual health, a gender transformative approach must be utilized when developing sexual health programs, services, policies, and interventions. Using a gender transformative approach can address and change the interventions that ignore or accommodate gender norms, relations, and structures and perpetuate harm by fostering risk‐taking, silencing debate, or depriving people of decision‐making power (Pederson et al., 2014).

Participants expressed that Black women's bodies are often not treated as worthy of protection or care. Perceptions of Black women as undeserving of protection are deeply rooted in the fabric of North American colonialism, society, and culture. This is showcased through early laws, such as practices that did not punish the sexual violation of Black women (Gross, 2018). It is also important to note that the perception of Black women's bodies as “masculine” dates from slavery because “strong” Black bodies could endure the brute force and produce more labor. This construction informs modern‐day perceptions of Black women's bodies (Slatton, 2012). Research shows that these perceptions impact Black women by influencing when or how health services and support are sought by and offered to Black women (Andrews et al., 2016). This unfortunate reality is highlighted in the belief that healthcare professionals often think Black women have higher pain thresholds. An article published in 2019 by Lee et al. found that Black people were 40% less likely to receive medication for pain and even less likely to receive opioids. This is in line with the study's findings; participants spoke of the perception of Black women's bodies as overtly “sexual” and how that leads to often being misdiagnosed with STIs and being denied proper healthcare. If unchallenged, the perceptions and stereotypes of Black women's bodies become preconceptions and generalizations that negatively affect them when faced with difficult situations in their daily lives, such as when accessing services (Stanton et al., 2022). This study contributes to the literature on the perception and hypersexualization of Black women's bodies by discussing how social constructs affect their ability to navigate sex and sexuality. The findings in this study highlight the gaps in the existing literature that solely focus on Black women's risky sexual behaviors and less on Black women's experiences navigating sex and sexual health. However, by having shared spaces of Black women discussing their sexual health and how they navigate sex, this can be achieved through increasing sexual self‐efficacy, encouraging sexual agency, providing space for Black women of marginalized communities, and through open discussions about sex positivity and pleasure in sexual health programming (Opara et al., 2021).

Participants expressed that women are often told not to have sex and are not given the proper tools to discuss their sexuality. Then, they are further shamed, stigmatized, or judged for having an interest in or engaging in sexual behaviors. Existing research supports this finding. Although men are allowed to have open discussions about sex, women are deemed responsible for sexual interaction and any unintended consequences, such as pregnancy (Fasula et al., 2007). Men can talk openly about sex, and discussions are often had without judgment and shame. Still, with young women, conversations around sex often revolve around shame and fear about virginity loss and pregnancy. Similar to previous research, the sexual double standard is often reinforced by parental figures (Axinn et al., 2011).

Mothers take a more proactive role in their son's sexuality and a more restrictive approach to their daughters' sexuality. Sons were often encouraged to buy and carry condoms, whereas daughters' sexual risk reduction attempts were often met with shame and judgment. Previous research discusses how the socialization of men and women concerning sex and sexuality may affect their ability to protect themselves from sexual risk (Wilson, 2011). This study showcases the apparent power imbalance between Black men and women regarding sex and navigating sexual health. Young Black women are often socialized to feel guilty about sex, whereas Black men are encouraged to engage in sexual relationships. The sexual double standards impact the self‐esteem of Black women, and Black women often have to work very hard to overcome the internalized shame of navigating sex and sexual health. When creating sexual health programming for Black women, it is essential to emphasize sex positivity and move away from the shame associated with sex. This will encourage positive discussions around sex and sexual health Hargons et al., 2020; Ware et al., 2019. A suggestion from participants in this study is that they would like to see programming and interventions that focused more on self‐pleasure and sexual satisfaction and less on Black women and their risky sexual behaviors. This is in line with findings from Opara et al. (2021) and Flowers (2018), which assert that failing to use a sex‐positivity framework when developing sexual health programming continues the trajectory of focusing on individual risk and removes focus from the more significant structural and sociocultural factors that impact sex and sexual health for Black women.

In keeping with the theme of gender roles, participants also noted that women entered transactional sexual relationships because men financially supported them, which often influenced their decisions regarding sexual negotiation and relationship‐seeking. This finding corresponds with the “Let's Talk About Sex for Money” study conducted by Wilson (Wilson & Flicker, 2017). The study found that young Black women often faced sexual objectification and low self‐esteem, which led to some young women becoming financially dependent on their partners. This points to a lack of sexual autonomy, which makes it harder to negotiate safer sex practices (Choi & Holroyd, 2007; Maher et al., 2013; Ranganathan et al., 2017). The study highlights transactional sex is a reality for some Black women, and when finances enter a relationship, the power dynamic becomes heightened. As Crooks et al. (2022) highlighted, interventions and programs targeted toward Black women should be multi‐level and focus on different aspects of health and well‐being. Health practitioners should ensure that sexual health programming encourages sexual autonomy and provides a space to speak openly about the power dynamics that may exist due to financial reasons and gender norms. Such programming should not only have sexual and reproductive health at its crux. However, it must also include other life skills, such as financial literacy and career counseling, which will aid in the empowerment of Black girls and women.

A plethora of evidence proves that Black bodies are disproportionately targeted by surveillance mechanisms (Gross, 2018; Leonard, 2009; Maynard, 2017). In *Dark Matters: On the Surveillance of Blackness*, Simone Browne examines surveillance of Black bodies by examining how surveillance technolo 2009 gies and practices that are racialized impact racialized bodies. Browne (2015) explains that surveillance historically and presently operates through the “white gaze” and discriminately targets the Black body. However, there is a lack of research that explicitly discusses Black women's experiences of surveillance while accessing sexual health services. Though they might not be surveilled in these spaces, ACB young women may feel they are because they grow up in a context where they are constantly being watched or expected not to engage in sexual activity. This finding is consistent with the “Let's Talk About Sex” (2011) study, in which young Black women discuss their unwillingness to access condoms in public spaces, such as community health centers or convenience stores because they constantly feel like they are being watched and scrutinized. It is essential to note that the surveillance “gaze” becomes internalized so that Black women navigate the world based on the feeling of being under constant surveillance. These young women and their sexualities might be surveilled by others such as mothers, fathers, aunties, and others, but they also might be surveilling each other because of the internalized gaze (Wilson, 2011). Young Black women would like sexual health programs and services that are free of judgment and can be accessed without fear of persecution. Intersectionality training for health practitioners that addresses racial and gender ideologies is suggested. This training should also include Black women from various walks of life to discuss their unique experiences. This training will address some environmental barriers to accessing sexual health programs and services for Black women. This method of incorporating intersectionality in practice has been seen in public health literature. In an articleby Heard and Wigginton (2023), intersectionality is utilized in sexual violence prevention training. Training includes face‐to‐face sessions that allow participants to reflect on their social positions, power, and privilege. The approach fosters an understanding of how social identity can influence service provision. Intentionally, incorporating an intersectional framework to training will help identify and address societal factors that contribute to providing sexual health services for young Black women.

## LIMITATIONS

Though this research study contributes to the literature on Black women and sexual health, a few limitations should be mentioned. The research study is centered on the discussion of sexual health and sexuality, which is a sensitive and highly stigmatized topic for many youths. Though several methods were employed to allow open discussion, the young women might have felt censored and uncomfortable speaking about issues related to sexual health. Another limitation is that the large majority of participants were cisgender and heterosexual women; this may have limited the analysis of gender roles because this group may have a narrower concept of gender roles. Lastly, a glaring shortcoming is the lack of representation of Black queer and trans women in this study. Though multiple attempts were made to generate an inclusive sample, the study is heteronormative and cisgender. As much as our team attempted to include the perspectives of queer and trans‐Black women in our participant pool, these spaces and the focus group format were probably not ideal for these subgroups. In addition to the lead author, Darko, facilitating both focus groups, our team also hired a queer‐identifying Black woman facilitator to support with recruitment and facilitation where needed. However, very few participants identified as queer or trans or chose not to disclose if they were, suggesting that perhaps the format of group discussions was less than ideal for garnering these diverse perspectives, given the stigma, transphobia, and homophobia that is still ever‐present.

The limitations of this study provide an opportunity for future studies to further explore and expand on this area of research. Future studies can focus on the reconceptualization of sexuality and sexual health for Black women, especially within sexual and reproductive programming. These studies could examine the stigma surrounding sexuality for Black women and focus on empowering Black women. These studies require researchers to examine the contextual, social, and political factors concerning sexual health, sexuality, and Black women. Future research can build upon this study and focus more on varying intersectional identities of Black women, such as religion, queer identities, immigration status, and disability; this will lead to a broader understanding of the nuanced experiences of Black women. There is a need and benefit for research that focuses on the social context of how diverse Black youth navigate sex and sexual health.

## CONCLUSION

This article contributes to the scant literature that studies the sexual health promotion of Black young women in Canada. Doing so fills a gap in the previous literature by adding an in‐depth perspective on the social factors that affect young Black women's experiences navigating sexual health. The results of this article are relevant because they highlight the importance of understanding the social factors that shape the lives and sexual health of many Black young women. As evidenced by the findings of this study, there are multiple layers of oppression that young Black women face, which impede their ability to navigate sexual health and sexuality safely. Young Black women often have to navigate their sexual health while feeling judged or surveilled due to the perceptions of Black women's bodies and sexuality. Future research can build upon this study and focus more on varying intersectional identities of Black women, such as religion, queer identities, immigration status, and disability; this will lead to a broader understanding of the nuanced experiences of Black women.

The conversations from the focus groups provide greater insight into Black women's experiences navigating sex and sexual health. The findings also provide greater insight into what Black women need in sexual health programming and services. This study has implications for sexual health programming and services targeting Black women because it highlights the nuanced experiences of being a Black woman. However, this article has limitations, such as lacking queer Black voices and other intersectional identities. Future researchers must use an intersectional lens when examining Black women and sexual health. Through the findings of this study, it is noted that Black women experience sex and sexual health differently than Black men and women of other ethnicities. The practical implications of this research include centering Black women's collective experiences and addressing the intersections of gender and race that impact their health. This study also adds to the existing literature that Black women are diverse in many facets of their identity but are connected by the fact that they experience discrimination as Black women (Prather et al., 2018). The Black women who participated in this study called for sexual health programming and services that focus on sexual health holistically, including various life skills in sexual health programming such as financial literacy, communication skills, and assertiveness training would be welcomed by Black women. Also, it was apparent that Black women needed spaces to talk openly about sex and sexual health without judgment or shame. Examining Black women's experiences navigating sex and sexual health through a structural and community‐based lens will lead to programming that strays away from the risky sex and sexual stereotypes that have befallen such programs in the past.

This study showcases the importance of involving young Black women in creating programs and services specifically targeted to them. The structural and sociocultural factors mentioned by the participants must be addressed when developing programming. Having Black youth involved in every facet of the planning and implementation is essential. Centering the voices of Black youth is crucial in changing the narrative regarding how young Black women navigate sex and sexual health. Young Black women wanted to be seen as more than the stereotypes placed on them. They want to be seen as fully human, cared for, and protected. Black women are layered and complex, they are mothers, sisters, and lovers too.

## Footnotes

1 Throughout this paper, we will use the acronym ACB to represent the diversity within Black communities in Canada. In particular, African communities represent the most recent wave of Black migration from continental Africa. Black‐Caribbean communities, which started to immigrate to Canada in sizeable numbers in the 1960s and 70s, and Black‐Canadian communities with connections to the underground railroad and Black Loyalist migration north of the USA−Canada border who have hundreds of years and several generations of history within the territorial borders of the Canadian nation‐state.

2 A woman is a person who identifies as a woman. This definition includes both cis‐gendered women (individuals born with biologically female sex organs, and who are assigned the gender of girl or woman at birth, and whose gender identity and expression socially match their assigned sex); and transgender women (born with sex organs that are defined as male, and who at some point over their life course identify as and gender express as a girl/woman). This definition acknowledges the importance of gender identity and expression in defining womanhood.

3 Due to the lack of race‐based data in Canada, this section will also draw upon literature from the United States and the United Kingdom that showcase comparable statistics regarding sexual health outcomes in Black women.
